# Resistance mechanisms and countermeasures in CLDN18.2-positive tumors: from molecular networks to clinical practice

**DOI:** 10.3389/fonc.2026.1910011

**Published:** 2026-08-26

**Authors:** Yutao Xia, Mingfang Wang, Taosheng Huang, Kai Sun, Peng Wang

**Affiliations:** Department of Oncology, Weifang Yidu Central Hospital, Weifang, Shandong, China

**Keywords:** CAR-T cell therapy, claudin 18.2, CLDN18.2, resistance mechanisms, targeted therapy, tumor microenvironment, zolbetuximab

## Abstract

Claudin 18.2 (CLDN18.2) has rapidly become a major target in solid-tumor oncology. Zolbetuximab, the first anti-CLDN18.2 antibody, was approved in 2024 for HER2-negative, CLDN18.2-high advanced gastric and gastro-esophageal junction adenocarcinoma, and more than one hundred CLDN18.2-directed trials are now under way across monoclonal antibodies, antibody-drug conjugates, bispecific antibodies and CAR-T cells. As these agents enter the clinic, resistance has become the central challenge. This review synthesizes the resistance mechanisms emerging from this experience and organizes them into six biological dimensions, separating those distinctive to CLDN18.2 from those shared across therapeutic platforms, and pairs each with mechanism-matched countermeasures weighted by level of evidence. From this synthesis we advance a central argument about treatment sequencing: because CLDN18.2 expression declines progressively under therapeutic pressure, continuous target-plus-chemotherapy acts on an eroding antigen substrate. For patients with high, homogeneous baseline expression, an early intensive induction followed by CLDN18.2-directed maintenance may therefore be preferable to indefinite target-plus-chemotherapy. We further consider the tissue-context-dependent biology of CLDN18.2 relevant to patient selection, and outline the prospective, biomarker-embedded trials needed to test these proposals.

## 1 Introduction

CLDN18.2 is one of the two splice isoforms of CLDN18 within the claudin tight-junction protein family. It is normally restricted to differentiated epithelial cells of the gastric mucosa (1, 2). During malignant transformation, loss of cell polarity disrupts the tight-junction localization of CLDN18.2. This exposes its first extracellular loop (ECL1) epitope on the cell surface and renders it accessible to therapeutic antibodies (3–5). Large immunohistochemistry studies demonstrate CLDN18.2 positivity in approximately 25 to 50% of gastric cancer (GC) cases (6–8), about 30% of pancreatic ductal adenocarcinomas (PDAC) (9, 10), and varying frequencies in esophageal adenocarcinoma and other gastrointestinal tumors (6, 11, 12). The target therefore combines broad tumor coverage with high specificity.

Zolbetuximab is a chimeric IgG1 monoclonal antibody targeting the ECL1 epitope of CLDN18.2 (13–15). When combined with mFOLFOX6 (a modified regimen of leucovorin, 5-fluorouracil, and oxaliplatin; SPOTLIGHT) or CAPOX (capecitabine plus oxaliplatin; GLOW), it improved progression-free survival (PFS) and overall survival (OS) in patients with HER2-negative, CLDN18.2-high advanced GC or gastroesophageal junction cancer (GEJC), and both improvements were statistically significant (16–18). It became the first agent directed against a novel antigen in GC since trastuzumab and the introduction of immune checkpoint inhibitor (ICI) therapy (5, 19, 20).

As zolbetuximab enters routine practice, resistance has become an immediate challenge. A substantial proportion of CLDN18.2-high patients show primary resistance, and most initial responders eventually progress within months (16, 17, 21–23). CLDN18.2 expression is also markedly heterogeneous in space and time: intratumoral heterogeneity is frequent and discordance between primary and metastatic lesions reaches about 25% (24–27). Serial-biopsy studies further show that roughly 40% of initially positive tumors lose CLDN18.2 positivity after a single chemotherapy cycle (28), and single-cell analyses link CLDN18.2-positive tumors to a distinct, chemotherapy-remodeled immunosuppressive microenvironment (28, 29). Together these findings indicate that resistance to CLDN18.2-targeted therapy is considerably more complex than initially anticipated.

At the same time, newer modalities have entered clinical evaluation. These include CAR-T cell therapies such as satricabtagene autoleucel (satri-cel, CT041) (30–32) and ADCs such as IBI343 (33), LM-302 (34), and sonesitatug vedotin (35, 36). They have also revealed distinct resistance mechanisms, engaging tumor microenvironment (TME)-mediated immunosuppression and intracellular signaling abnormalities that further complicate the therapeutic landscape. This review pursues three integrated objectives. First, we map resistance to CLDN18.2-targeted therapy across six biological dimensions, separating three CLDN18.2-distinctive vulnerabilities from three platform-shared resistance modes whose expression in CLDN18.2 contexts has distinctive kinetics. To our knowledge, this distinction has not been drawn explicitly in prior CLDN18.2-targeted reviews (5, 22, 37–39); **Table 1** contrasts the scope, organizing principle, and distinctive features of the present review with those earlier syntheses. Second, we propose mechanism-matched countermeasures whose translational priority is shaped by which resistance class they target, with each recommendation weighted by epistemic status. Third, and most consequentially for clinical practice, we draw on the consistent observation that CLDN18.2 expression is progressively lost under treatment pressure to argue that the optimal use of these agents may depend on treatment sequencing: an intensive multi-agent induction delivered during the early, high-antigen window, followed by CLDN18.2-directed maintenance monotherapy, rather than indefinite continuous target-plus-chemotherapy. As a complementary consideration for patient selection, we also synthesize the tissue-context-dependent biology of CLDN18.2 across tumor types.

**Table 1:** The present review versus prior CLDN18.2 reviews.

Review (year)	Scope	Organizing principle	Resistance coverage	Sequencing / maintenance thesis	Pan-cancer context
Nakayama et al., 2024 (5)	CLDN18.2 as target (overview)	By target / agent	Partial	None	Limited
Dominguez Wiscovitch et al., 2025 (22)	CLDN18.2 in GI cancers	By tumor type / modality	Limited	None	Partial
Yamamoto et al., 2026 (38)	Emerging biologic agents	By modality	Moderate	None	Limited
Li et al., 2026 (39)	ADCs in gastric cancer	By ADC resistance mechanism	ADC-focused	None	None
Present review	Pan-cancer CLDN18.2 resistance and countermeasures	Six-dimension mechanism framework (3 distinctive + 3 shared)	Comprehensive, across 4 modalities	Yes: depleting-target to induction-and-maintenance	Yes: tissue-specific functional polarity

ADC, antibody-drug conjugate; CLDN18.2, claudin 18.2; GI, gastrointestinal. The present review is distinguished by a six-dimension resistance framework separating three CLDN18.2-distinctive vulnerabilities from three platform-shared modes, an induction-and-maintenance sequencing thesis grounded in progressive antigen loss, and explicit evidence-level weighting of each recommendation.

## 2 Biological basis of CLDN18.2

CLDN18.2 is one of two alternatively spliced isoforms of CLDN18 on chromosome 3q22.3; in normal tissue it is exquisitely restricted to the chief and parietal cells of the gastric fundic glands (1, 2). This extreme tissue specificity is the cornerstone of its value as a therapeutic target.

Aberrant CLDN18.2 expression in tumors takes two forms. The first is mislocalization from the lateral membrane to the free apical surface, and the second is transcriptional upregulation (1, 3, 40). Both forms lead to ECL1 epitope exposure (3, 4). Immunohistochemical data show CLDN18.2 positivity in approximately 25 to 50% of GC (6), about 30% of PDAC (9), about 30% of esophageal adenocarcinoma (41), about 3% of colorectal cancer enriched in MMR-deficient or BRAF V600E-mutated subtypes (11), about 8% of lung adenocarcinoma (42), and about 70% of cervical gastric-type adenocarcinoma (43).

CLDN18.2 performs non-uniform biological functions across tumor types, and these functions can appear paradoxical. In GC, high CLDN18.2 expression is associated with diffuse-type histology in the Lauren classification, which generally carries a worse prognosis (44, 45). The functional role of CLDN18.2 in GC remains context-dependent: while claudins can act as either tumor promoters or suppressors, recent evidence indicates that CLDN18.2 knockdown suppresses GC cell proliferation, pointing to a tumor-promoting role (46, 47). Similarly, in PDAC, a ZIC2-CLDN18.2-ERK1/2 signaling axis promotes tumor proliferation, migration, and invasion (48). In lung adenocarcinoma, strong CLDN18.2 positivity is associated with more favorable outcomes, particularly in EGFR wild-type and PD-L1 below 1% double-negative subgroups (42). This context-dependent function profoundly shapes how CLDN18.2 should be understood as a therapeutic target across cancer types.

A recent siRNA knockdown study showed that CLDN18.2 forms an essential complex with ZO-1 that maintains tight-junction integrity in gastric cancer cells; silencing CLDN18.2 downregulates ZO-1, occludin and β-catenin, reduces transepithelial electrical resistance, and enhances migration, invasion and metastatic nodule formation in xenografts (49). These findings position CLDN18.2 as an epithelial-barrier gatekeeper, providing a molecular basis for understanding how CLDN18.2 loss may promote chemotherapy resistance through impaired drug penetration.

## 3 Landscape of CLDN18.2-targeted therapy

Zolbetuximab is a recombinant chimeric IgG1 monoclonal antibody that specifically binds the ECL1 epitope of CLDN18.2 (13). It exerts antitumor effects mainly through antibody-dependent cellular cytotoxicity (ADCC) and complement-dependent cytotoxicity (CDC) (3, 37). Its key feature is selective binding to CLDN18.2 exposed on tumor cells that have lost polarity, rather than to normal polarized gastric mucosa. On-target, off-tumor gastric toxicity is nonetheless observed clinically.

SPOTLIGHT (mFOLFOX6 backbone, NCT03504397) (16) and GLOW (CAPOX backbone, NCT03653507) (17) were Phase 3 randomized controlled trials. Both enrolled patients with HER2-negative, CLDN18.2-high advanced GC or GEJC, defined as at least 75% of tumor cells showing moderate-to-strong (2+/3+) membranous staining by the VENTANA CLDN18 (43-14A) assay. Both trials consistently showed that zolbetuximab plus chemotherapy significantly prolonged median PFS (GLOW 8.21 vs. 6.80 months; SPOTLIGHT 10.61 vs. 8.67 months) and median OS (GLOW 14.39 vs. 12.16 months; SPOTLIGHT 18.23 vs. 15.54 months), with statistically significant hazard ratios (16, 17). On this basis, zolbetuximab received Japan PMDA approval in March 2024, US FDA approval in October 2024, and European Commission marketing authorization in September 2024 (19, 20).

A total of 78 trials are currently active or recruiting, which reflects the dynamism of the field. Among them, 14 Phase 3 randomized controlled trials now cover all four major therapeutic modalities: monoclonal antibodies, ADCs, bispecific antibodies, and CAR-T cell therapy.

CLDN18.2-directed ADC Phase 3 trials have emerged in a concentrated burst (**Table 2**). In the second-line GC setting, AZD0901 (AZD0901, CMG901 and the INN sonesitatug vedotin denote the same Astellas/AstraZeneca molecule, with the trial-specific designation retained in each instance) (50) (NCT06346392), IBI343 (33) (NCT06238843), LM-302 (34) (NCT06351020), JS107 (51) (NCT07284134) and BL-M05D1 (52) (NCT07518147) compete against standard chemotherapy. The largest CLDN18.2 trial to date is the first-line, multi-arm Phase 3 study of sonesitatug vedotin (NCT07431281; n=2,130), which tests the ADC alone or with the bispecific rilvegostomig against nivolumab- and zolbetuximab-based chemotherapy. LM-302 plus tislelizumab plus CAPOX (NCT07385703) is recruiting in the first line, and IBI343 is in the second-line-plus G-HOPE-002 pancreatic study (NCT07066098).

**Table 2:** Key phase 3 clinical trials of CLDN18.2-targeted therapies (as of April 2026).

NCT number	Drug / regimen	Modality	Line	Tumor type	N	Status / primary completion	Resistance dimension addressed*
NCT03504397	Zolbetuximab + mFOLFOX6 (SPOTLIGHT)	mAb	1L	GC/GEJC	565	Completed	Baseline antigen-targeted; ADCC/CDC
NCT03653507	Zolbetuximab + CAPOX (GLOW)	mAb	1L	GC/GEJC	507	Completed	Baseline antigen-targeted; ADCC/CDC
NCT06901531	Zolbetuximab + Pembrolizumab + Chemo	mAb + ICI	1L	GC/GEJC	500	Recruiting; 2028-09	Immunosuppressive TME (PD-L1 co-expr.; EBV/MSI)
NCT06177041	M108 + CAPOX	mAb	1L	GC/GEJC (PD-L1 CPS<5)	486	Recruiting; 2027-01	Antigen heterogeneity; PD-L1-low subgroup
NCT07431281	Sonesitatug vedotin ± Rilvegostomig	ADC	1L	GC/GEJC/EAC	2130	Recruiting; 2029-08	Antigen heterogeneity (bystander); ICI resistance (36)
NCT06346392	AZD0901 (CMG901) vs. SoC	ADC	2L+	GC/GEJC	572	Recruiting; 2026-05	Acquired antigen loss; autophagy escape† (75)
NCT06238843	IBI343 vs. SoC	ADC	2L+	GC/GEJC	450	Recruiting; 2026-12	Acquired antigen loss; autophagy escape† (75)
NCT06351020	LM-302 vs. Apatinib / Irinotecan	ADC	2L+	GC/GEJC	387	Active; 2026-11	Acquired antigen loss; heterogeneity (bystander) (36)
NCT07385703	LM-302 + Tislelizumab + CAPOX	ADC + ICI	1L	GC/GEJC	752	Not yet recruiting; 2028-10	ICI resistance in MSS (ICD via payload)† (76)
NCT07284134	JS107 vs. SoC	ADC	2L+	GC/GEJC	560	Recruiting; 2027-10	Acquired antigen loss; post-zolbetuximab†
NCT07518147	BL-M05D1 vs. SoC	ADC	2L+	GC/GEJC	438	Not yet recruiting; 2029-12	Acquired antigen loss; post-zolbetuximab†
NCT07066098	IBI343 vs. Placebo (G-HOPE-002)	ADC	2L+	Pancreatic	201	Recruiting; 2027-06	PDAC: ZIC2-ERK1/2 axis; MDSC barrier† (48)
NCT07079228	QLS31905 + Nab-P + Gemcitabine	Bispecific	1L	Pancreatic	602	Recruiting; 2028-12	PDAC: MDSC immunosuppression; T-cell redirection
NCT07103668	CLDN18.2 CAR-T vs. Control	CAR-T	Locally adv.	Locally advanced GC	150	Recruiting; 2027-05	TME immunosuppression (IDO1 axis); infiltration barrier (68)

mAb, monoclonal antibody; ADC, antibody-drug conjugate; ICI, immune checkpoint inhibitor; GC, gastric cancer; GEJC, gastroesophageal junction cancer; EAC, esophageal adenocarcinoma; SoC, standard of care; ICD, immunogenic cell death; TME, tumor microenvironment; MDSC, myeloid-derived suppressor cell. *The “Resistance Dimension Addressed” column reflects the authors’ mechanistic interpretation, not the trials’ official endpoints. †Denotes a hypothesized rather than prespecified mechanism. The 'Resistance Dimension Addressed' column reflects the authors' mechanistic framework (Sections 4-5) rather than each trial's pre-specified endpoints; †denotes a hypothesised mechanism not formally tested by the listed trial. Bracketed numbers refer to the mechanistic references in the main text.

The four platforms differ in where their resistance risk concentrates: monoclonal antibodies and ADCs are limited chiefly by antigen density and heterogeneity, bispecific antibodies by chronic CD3-driven T-cell exhaustion and antigen modulation, and CAR-T cells by solid-tumor infiltration and the immunosuppressive microenvironment. **Table 3** summarizes the principal advantages, limitations, and dominant resistance mechanism of each modality and maps them to the resistance dimensions dissected mechanism-by-mechanism in Section 4.

**Table 3:** CLDN18.2-targeted therapeutic modalities: advantages, limitations, and dominant resistance mechanisms.

Modality (mechanism)	Representative agents	Principal advantages	Principal limitations	Platform-specific dominant resistance	Resistance dimension (Section 4)
Monoclonal antibody (ADCC/CDC)	Zolbetuximab	First approved; manageable toxicity; validated companion diagnostic	Depends on antigen density and effector cells; on-target gastric toxicity	Antigen loss/density decline; immunosuppressive TME	4.1, 4.4
Antibody-drug conjugate	AZD0901/CMG901, IBI343, LM-302	Bystander effect covers heterogeneous antigen; payload independent of host immunity	On-/off-tumor toxicity; payload-class dependence	Autophagy-mediated payload escape; antigen loss; payload-class efficacy ceiling	4.1, 4.5
Bispecific antibody (CD3 / 4-1BB)	Givastomig, AZD5863, IBI389	Off-the-shelf; recruits polyclonal T cells; conditional (tumor-restricted) activation	Broad T-cell exhaustion; accentuated antigen modulation; CRS	Chronic CD3-driven exhaustion; antigen internalization/downregulation	4.6
CAR-T cell	Satri-cel (CT041)	Deep responses; engineerable (armored / logic-gated)	Poor solid-tumor infiltration; TME suppression; limited persistence	Infiltration/polarity barrier; IDO1/TME; trogocytosis-beta-catenin	4.2, 4.3.3, 4.4

ADC, antibody-drug conjugate; ADCC, antibody-dependent cellular cytotoxicity; CAR-T, chimeric antigen receptor T cell; CDC, complement-dependent cytotoxicity; CD3, cluster of differentiation 3; CRS, cytokine release syndrome; IDO1, indoleamine 2,3-dioxygenase 1; TME, tumor microenvironment. The table maps each platform to the resistance dimensions dissected mechanism-by-mechanism in Section 4.

## 4 Resistance mechanisms of CLDN18.2-targeted therapy

**Figure 1:**
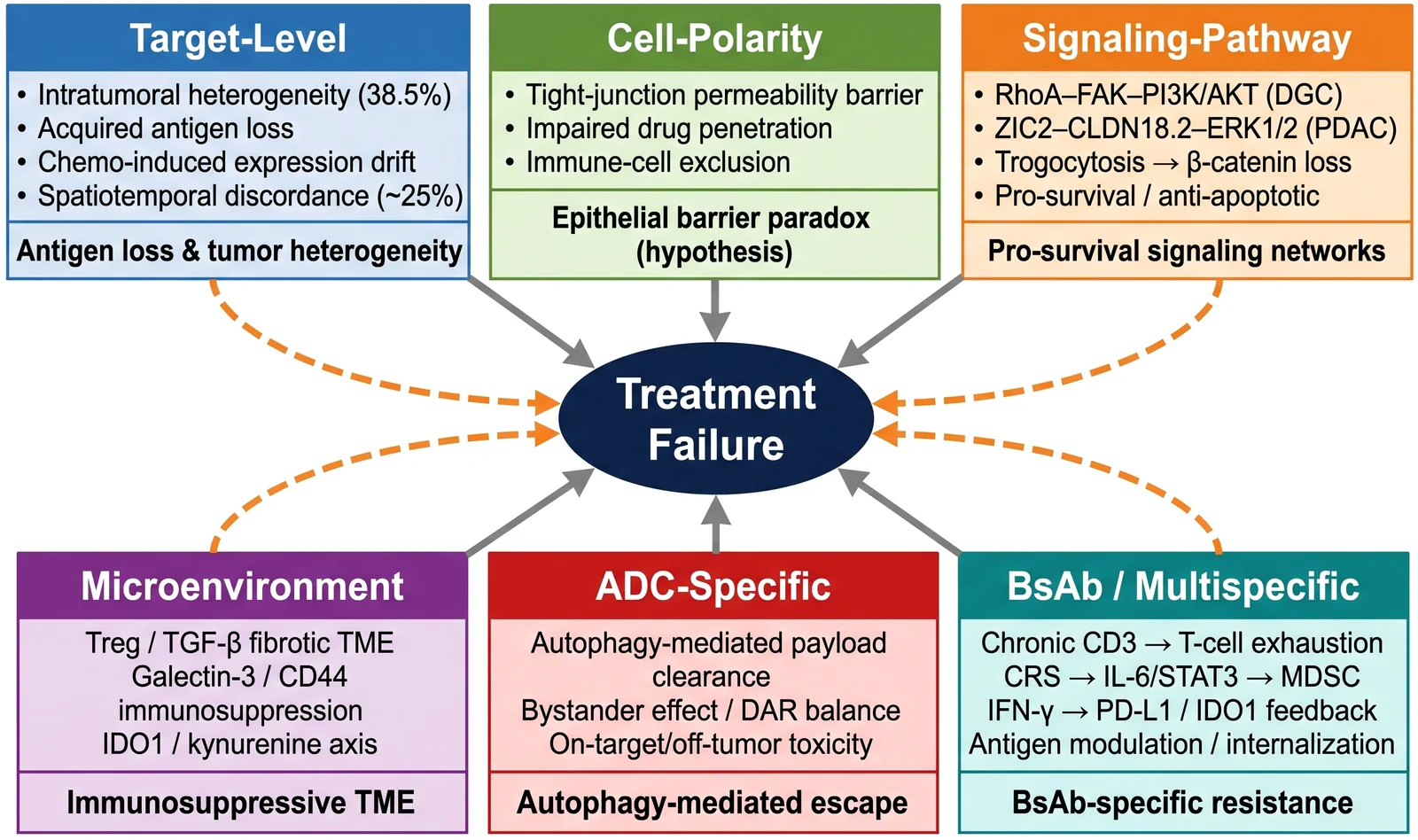
The multidimensional resistance network of CLDN18.2-targeted therapy.

Resistance to CLDN18.2-targeted therapy can be classified as primary or acquired. Based on the current literature, the mechanisms can be organized across six dimensions, shown in **Figure 1**. These are target-level antigen loss and heterogeneity, cell-polarity-related barriers, pro-survival signaling networks, the immunosuppressive microenvironment, ADC-specific autophagy escape, and mechanisms specific to bispecific and multispecific antibodies.

### 4.1 Target-level resistance: antigen loss and tumor heterogeneity

#### 4.1.1 Intratumoral heterogeneity

An analysis of 166 resected metastatic GC specimens found intratumoral heterogeneity in 38.5%, with about 25.2% of patients showing discordant positivity across lesions (27); liver metastases were significantly less positive and concordant with the primary than peritoneal metastases. EBV-positive tumors show higher positivity (up to 70.2%) than EBV-negative (about 29.4%) (53), and such antigen-density differences across molecular subtypes may affect ADCC-based therapy.

#### 4.1.2 Acquired antigen loss

Secondary loss of CLDN18.2 expression during treatment has been documented in multiple case reports and cohort studies (54, 55). One case report described a young female patient who progressed on zolbetuximab (21). Her primary tumor and bone-marrow metastases showed strong, diffuse CLDN18.2 expression, but a concurrently sampled Krukenberg tumor was completely CLDN18.2-negative. This suggests that clonal selection pressure may drive differential antigen escape at different metastatic sites. The phenomenon closely resembles HER2 loss following trastuzumab treatment in HER2-positive breast cancer (56), and it emphasizes the importance of repeat biopsy with CLDN18.2 reassessment at progression.

Acquired antigen loss after targeted therapy is not unique to CLDN18.2; the CD19-directed therapy literature in B-cell acute lymphoblastic leukemia (B-ALL) is the most extensively characterized precedent. Approximately 10–20% of pediatric responders to CD19 CAR-T cell therapy experience CD19-epitope-loss relapse (57). Three escape routes, described across separate primary reports rather than within any single paper, are now firmly established. First, convergent acquired mutations and alternative splicing of CD19 enable expression of N-terminally truncated CD19 isoforms lacking the FMC63 exon-2 epitope, rendering tumor cells invisible to CART-19 (57). Second, lineage switch to a myeloid phenotype has been reported in KMT2A-rearranged leukemia (58). Third, CAR-T-mediated trogocytosis transfers CD19 from tumor cells onto T cells, simultaneously producing reversible, post-transcriptional antigen-low tumor escape and promoting fratricidal CAR-T killing plus exhaustion of the surviving CAR-T pool (59). The CLDN18.2 setting differs in two important respects. First, no CLDN18.2 isoform-skipping splice variant analogous to CD19 exon-2 has yet been described, and conservation of the ECL1 epitope across tumor types suggests structural constraint. Second, the trogocytosis pathway invoked in CLDN18.2 drives a downstream β-catenin–CXCL12 cascade and systemic immune-senescence phenotype (60) that is functionally distinct from the antigen-clearing, fratricide-driven trogocytosis described for CD19 CAR-T (59). The CD19 experience nevertheless suggests three concrete priorities for CLDN18.2-targeted programs: (i) serial post-progression biopsy with full-transcriptome sequencing to detect CLDN18 splice variants or expression-rescue mutations; (ii) flow-based screening for trogocytosis-mediated CLDN18.2 transfer onto infused CAR-T cells, paired with CAR-T persistence kinetics; and (iii) early development of dual-antigen CAR-T strategies (CLDN18.2/MSLN, CLDN18.2/NKG2D-ligand), drawing on the CD19/CD22 dual-target precedent in relapsed pediatric B-ALL, where coadministration of CD19- and CD22-directed CAR-T achieved a 99% CR rate and only 1/43 relapses involved CD22 loss versus 17/43 with CD19 loss (61).

#### 4.1.3 Chemotherapy-induced dynamic expression changes

A prospective single-arm Phase 2 trial performed serial biopsy analyses in 57 advanced GC patients who underwent sequential capecitabine and oxaliplatin chemotherapy followed by pembrolizumab (28). Baseline CLDN18.2 positivity was 40.4%. After one cycle of chemotherapy, 40% of initially positive patients lost CLDN18.2 expression, while 10% of initially negative patients acquired expression, and this acquisition was more common in tumors with higher stromal fibrosis. Single-cell analysis indicated that CLDN18.2-positive tumors harbor stronger TGF-β signaling, more matrix-remodeling activity, and greater regulatory T-cell infiltration. This immunosuppressive, fibrotic TME may regulate CLDN18.2 expression through epigenetic mechanisms, which would provide a molecular basis for chemotherapy-induced expression drift.

Across these serial-biopsy studies the direction of the effect is consistent: therapeutic pressure predominantly reduces CLDN18.2 positivity (28, 54, 55). The magnitude, however, varies widely, because studies differ in scoring threshold and antibody clone (for example VENTANA CLDN18 43-14A 2+/3+ in at least 75% of cells versus alternative cut-offs), in tumor stage, and in sampling time-point and site (7, 27). Assay standardization is therefore the dominant source of apparent inconsistency (24). The qualitative consistency itself supports the view that CLDN18.2 is a dynamic target requiring serial reassessment, while the quantitative spread underscores the need for harmonized companion diagnostics.

### 4.2 Cell-polarity-level resistance: a chemotherapy-penetration barrier

In normally polarized epithelial cells, CLDN18.2 maintains tight-junction integrity through direct physical interaction with ZO-1 and controls paracellular permeability (40, 49, 62). Aberrant CLDN18.2 expression in tumor cells makes them targetable (5), but it may also preserve a degree of tight-junction barrier function. That residual barrier could restrict the intratumoral penetration and accumulation of chemotherapeutic agents, particularly large-molecule drugs and hydrophilic platinum compounds. We propose that this constitutes a structural chemoresistance barrier in CLDN18.2-high tumors.

When tumor cells lose CLDN18.2 expression entirely, the further disruption of cell polarity may paradoxically enhance tumor invasiveness and aggressiveness (49). This could help explain the more aggressive phenotype observed after acquired resistance. CLDN18.2-regulated paracellular permeability may also influence how efficiently immune cells infiltrate the tumor parenchyma. Such an effect would be related to the immune-excluded TME phenotype and would partly explain the insufficient CAR-T cell infiltration seen in CLDN18.2-positive solid tumors (63).

This hypothesis must be weighed against an important counter-observation. On-target, off-tumor gastric toxicity, seen as nausea and vomiting with zolbetuximab and as gastrointestinal adverse events with CLDN18.2-directed ADCs and CAR-T cells, indicates that therapeutic antibodies and cells do reach CLDN18.2 on normally polarized gastric mucosa, where tight junctions are intact (16, 17, 30, 64). Epitope accessibility is therefore not fully abolished by preserved polarity, and the barrier we propose is best framed as a quantitative modifier of intratumoral drug penetration and effector access rather than an absolute shield. The proposal consequently predicts a graded, not binary, effect: penetration of large or hydrophilic agents should correlate inversely with residual junctional integrity, a prediction directly testable by pairing CLDN18.2/ZO-1 co-staining with intratumoral drug-level or effector-infiltration measurements. We therefore flag this as a hypothesis requiring falsification rather than an established mechanism.

### 4.3 Signaling-pathway-level resistance: pro-survival networks

#### 4.3.1 RhoA-FAK-PI3K/AKT pathway

**Figure 2:**
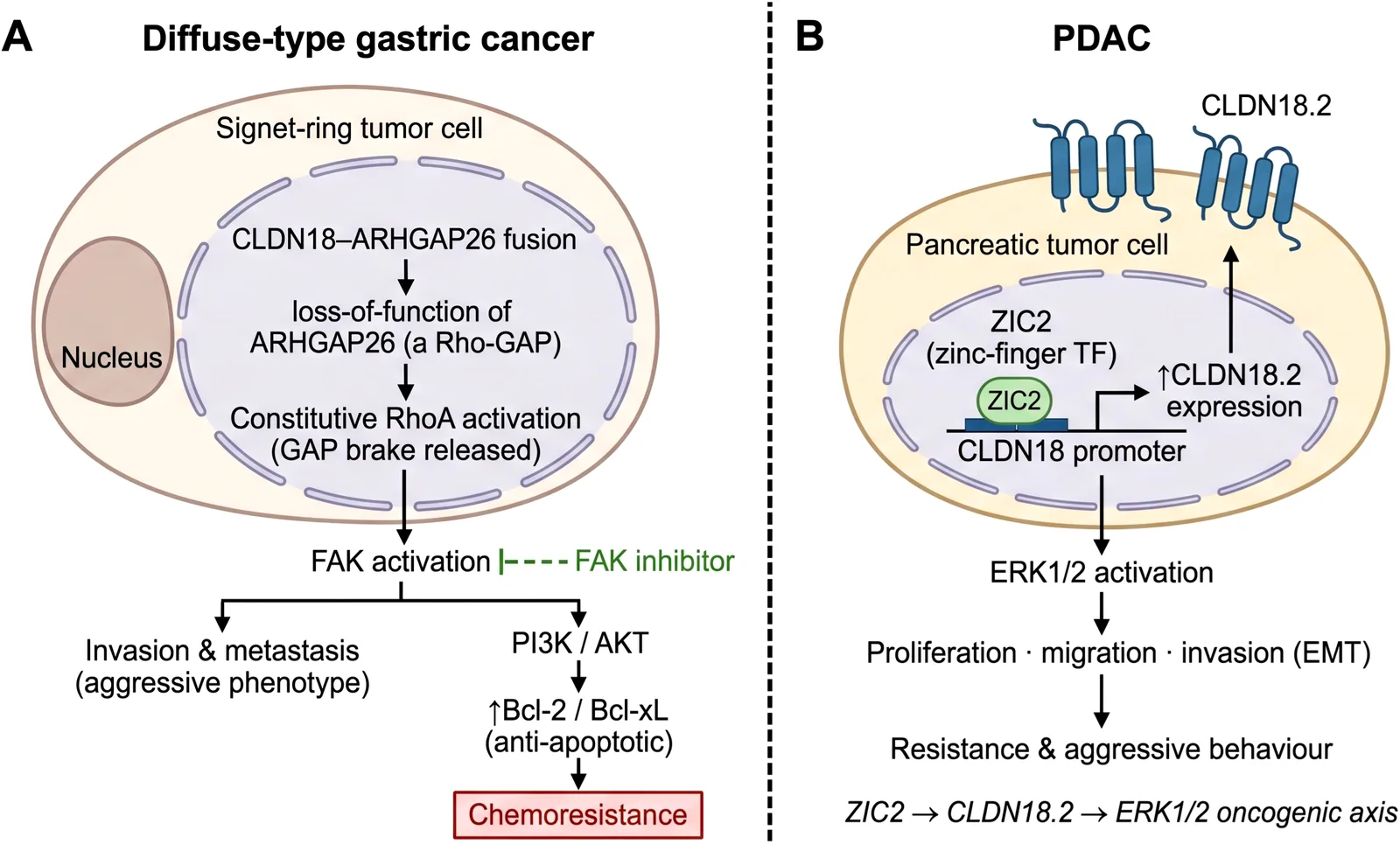
Context-dependent pro-survival signaling linked to CLDN18.2.

Diffuse-type gastric cancer (DGC), the histological subtype with the highest CLDN18.2 enrichment, is also enriched for CLDN18-ARHGAP26 gene fusions (65, 66). The fusion alters the function of ARHGAP26 (a Rho-GAP), disrupting its normal negative regulation of RhoA; the resulting RhoA-FAK axis promotes invasion and metastasis, and FAK-driven PI3K/AKT signaling up-regulates Bcl-2 and Bcl-xL to confer chemoresistance (65) (**Figure 2A**). FAK inhibitors have shown preclinical antitumor efficacy in DGC models (66), suggesting that combining CLDN18.2-targeted therapy with FAK inhibition may be synergistic in the diffuse-type subgroup.

#### 4.3.2 ERK1/2 pathway

In PDAC, the pro-tumorigenic role of CLDN18.2 is mediated through ZIC2, a zinc-finger transcription factor. ZIC2 activates CLDN18.2 expression by binding the CLDN18 promoter, and CLDN18.2 in turn activates the ERK1/2 pathway to enhance proliferation, migration, and invasion (48). This ZIC2-CLDN18.2-ERK1/2 axis (**Figure 2B**) may be a partial driver of chemoresistance in pancreatic cancer. It also raises the possibility that CLDN18.2-targeted therapy in PDAC exerts additional antitumor effects by disrupting this oncogenic axis, although this hypothesis awaits clinical validation. The axis derives from a single discovery cohort and has not yet been independently replicated in additional PDAC datasets; mechanism-matched recommendations to disrupt it via CLDN18.2 targeting should weight this evidence accordingly.

#### 4.3.3 β-catenin/Wnt pathway

**Figure 3:**
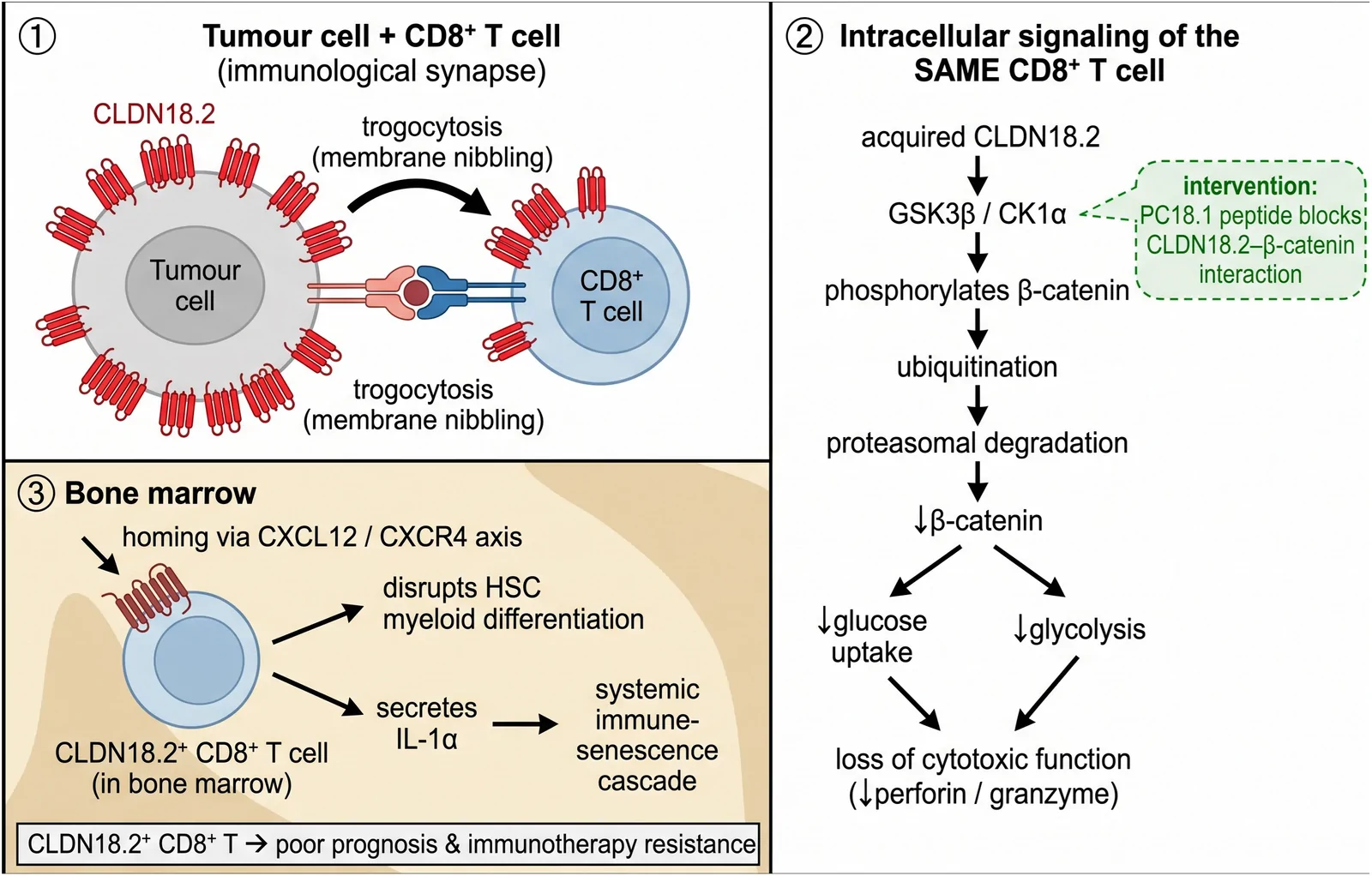
Trogocytosis-driven CLDN18.2 transfer reprograms CD8+ T cells and seeds systemic immune senescence.

A recent study defined a novel CLDN18.2-mediated immune-evasion mechanism in pancreatic cancer (**Figure 3**) (60). Through trogocytosis, CD8+ T cells acquire CLDN18.2 from tumor cells; surface CLDN18.2 then drives GSK3β/CK1α-mediated phosphorylation, ubiquitination and proteasomal degradation of β-catenin, suppressing glucose uptake and glycolysis and inactivating cytotoxic function. These CLDN18.2-positive CD8+ T cells home to bone marrow via the CXCL12/CXCR4 axis, disrupt myeloid differentiation of hematopoietic stem cells, and induce a systemic immune-senescence cascade through IL-1α. Clinically, their presence correlated with poor prognosis and immunotherapy resistance, and a peptide disrupting the CLDN18.2–β-catenin interaction reversed the phenotype in mice. Confirming the generality and therapeutic interpretability of this trogocytosis–β-catenin circuit is now a priority; systemic monitoring strategies are discussed later.

### 4.4 Microenvironment-level resistance: the immunosuppressive TME

#### 4.4.1 TME characteristics and immunosuppression in CLDN18.2-positive tumors

A systematic multiplex immunohistochemistry analysis showed that CLDN18.2-positive GC has a distinct microenvironment (67). These tumors show increased infiltration by CD8+ T cells and CD66b+ neutrophils, together with a higher proportion of M1-type macrophages. The result is paradoxical. Despite a larger subset of exhaustion-marker-negative CD8+ T cells, these tumors show suppressed antitumor immunity, and this translates into significantly poorer clinical outcomes, with a median immune-related OS (irOS) of 10.03 versus 20.13 months (p = 0.044). Prospective single-cell RNA sequencing further showed enrichment of regulatory T cells (Tregs) and of galectin-3/CD44 signaling, a pathway that promotes immunosuppression and stromal fibrosis (28). This may account for the limited response rates to combined chemotherapy plus immunotherapy in CLDN18.2-positive patients.

Among these immunosuppressive circuits, the tryptophan-kynurenine/IDO1 axis is currently the best supported as a driver of resistance: it is the only node in the CLDN18.2 setting with combined mechanistic, causal, and measurable-biomarker evidence, since IDO1 inhibition functionally rescues CLDN18.2 CAR-T activity (68) through a well-defined AhR-mediated program (69) that yields a quantifiable serum kynurenine-to-tryptophan readout. The Treg/TGF-β and fibrotic-stroma axis is next, with strong single-cell and multiplex association but largely correlative evidence (28, 67); the trogocytosis-driven β-catenin/marrow-senescence axis is mechanistically novel and includes a murine reversibility experiment but rests on a single foundational study awaiting replication (60); and the neutrophil (NE-3/NLR) association is single-center and preliminary (70). We therefore prioritize the IDO1 axis for prospective combination testing and pharmacodynamic monitoring.

#### 4.4.2 IDO1 and the tryptophan-kynurenine metabolic axis

**Figure 4:**
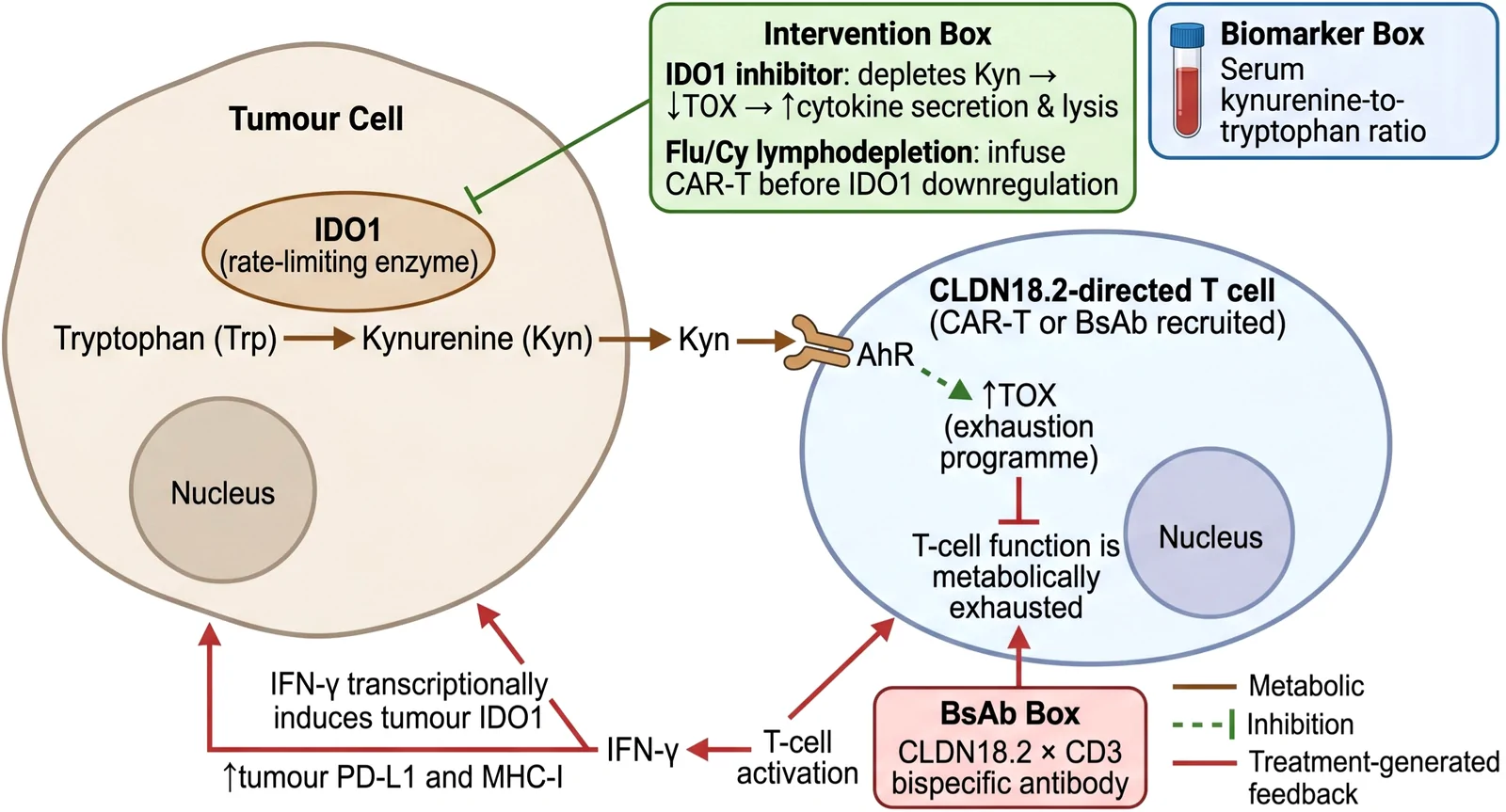
The IDO1–kynurenine axis as a constitutive and treatment-induced resistance node.

Indoleamine 2,3-dioxygenase 1 (IDO1), the rate-limiting enzyme of tryptophan catabolism, and the related enzyme tryptophan-2,3-dioxygenase (TDO) both generate kynurenine, which suppresses T cells and induces exhaustion via aryl-hydrocarbon-receptor signaling (**Figure 4**) (69). In gastrointestinal cancer models, IDO1 inhibition depleted kynurenine, downregulated exhaustion factors such as TOX, and enhanced CLDN18.2 CAR-T cytokine secretion and tumor-cell lysis (68); a comparable IDO1-dependent benefit has also been shown for T-cell-engaging bispecific antibodies in other tumor types (71); fludarabine/cyclophosphamide lymphodepletion itself downregulates TME IDO1, creating a favorable metabolic niche for infused CAR-T. The axis is not limited to constitutive suppression; it also emerges as a treatment-generated mechanism under bispecific-antibody pressure. The on-treatment serum kynurenine-to-tryptophan ratio may therefore serve as a monitoring biomarker, and we propose co-administration of an IDO1 inhibitor in next-generation combinations.

The CLDN18.2-related immunosuppressive TME shares architecture with the broader pancreatic, biliary, and diffuse-type gastric cancer microenvironment, and the prior decade of TME-directed therapeutic attempts in those settings is instructive. Heterologous prime/boost vaccination with GVAX pancreas and CRS-207, a Listeria-vectored mesothelin vaccine, achieved a statistically significant but modest OS gain in metastatic pancreatic adenocarcinoma (6.1 vs 3.9 months; HR 0.59; P = 0.02), illustrating that single-modality TME-directed immunotherapy can move the needle without delivering transformative benefit (72). The COMBAT phase IIa trial of the CXCR4 antagonist BL-8040 with pembrolizumab ± NAPOLI-1 chemotherapy in metastatic PDAC generated hypothesis-generating signals (cohort 2 ORR 32%, DCR 77%) but was a single-arm study explicitly calling for randomized confirmatory trials; CXCR4 antagonism has not since established a clear Phase 3 advance in PDAC (73). Checkpoint blockade combined with chemotherapy has fared no better: nivolumab + nab-paclitaxel + gemcitabine in a phase 1 advanced-PDAC trial produced a modest single-arm signal (ORR 18%, mPFS 5.5 mo, mOS 9.9 mo) that did not exceed historical nab-paclitaxel/gemcitabine benchmarks, and the investigators concluded the results did not support further investigation (74). The shared lesson across these platform-level disappointments is that monotargeting a single immunosuppressive node, whether by vaccination, chemokine blockade, or checkpoint inhibition, rarely succeeds in stromal-rich solid tumors, because the TME compensates through parallel circuits. The implication for CLDN18.2 combinations is structural rather than tactical: any added TME-targeted agent should be positioned as a niche-clearing adjuvant to a CLDN18.2-directed cytotoxic backbone that itself drives the dominant anti-tumor action, not as a stand-alone immune-restorative agent. Engineered CLDN18.2 CAR-T designs that embed microenvironment modification within the cytotoxic cell itself implicitly adopt this positioning, whereas stand-alone TME drugs added to CLDN18.2 therapy should be required to demonstrate combination benefit beyond what the cytotoxic backbone alone delivers, rather than being credentialed on preclinical mechanism alone.

### 4.5 ADC-related resistance: autophagy-mediated escape

**Figure 5:**
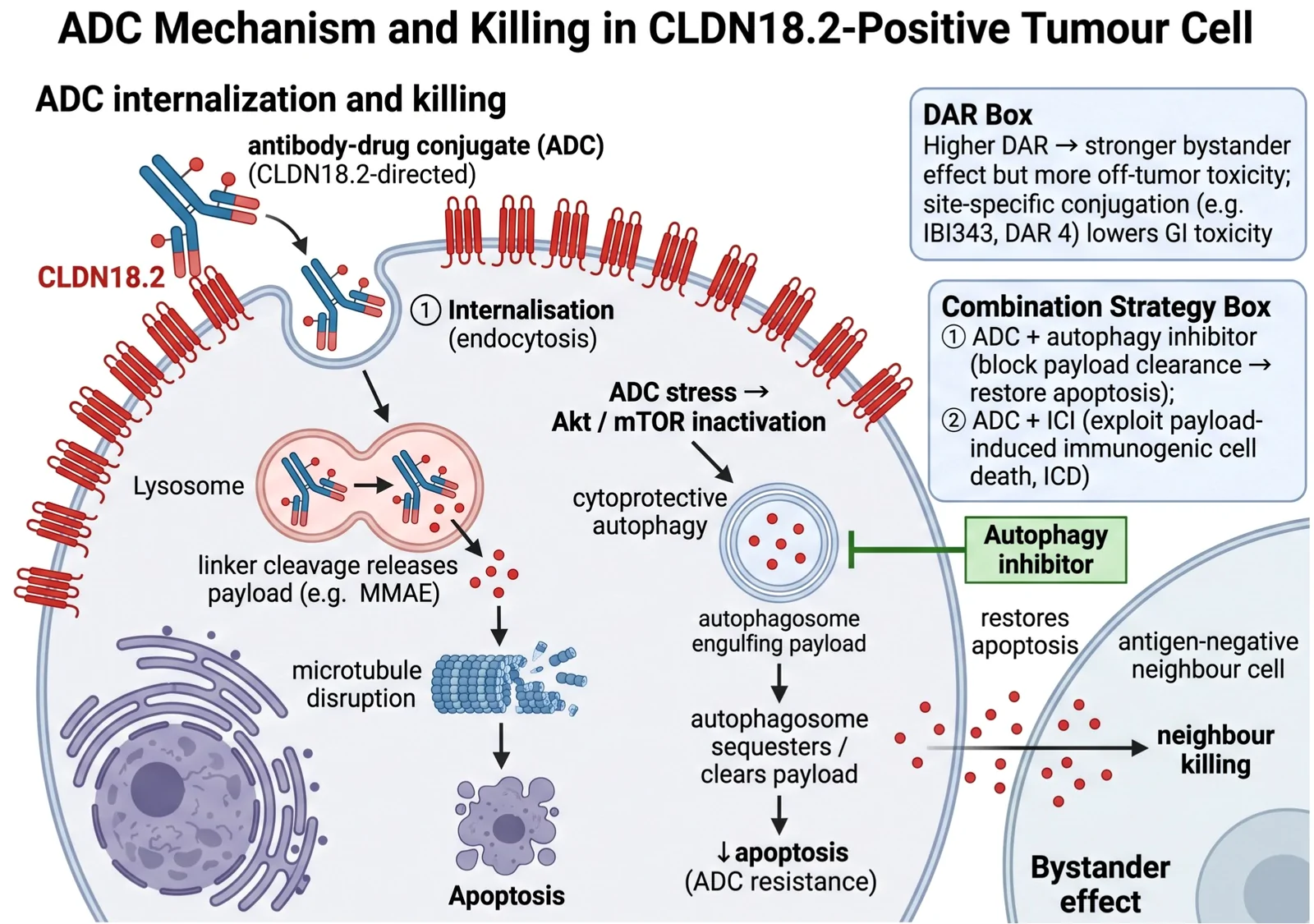
ADC killing versus autophagy-mediated escape in CLDN18.2-positive tumor cells.

The resistance mechanisms of ADCs differ substantially from those of conventional monoclonal antibodies and CAR-T cells. An ADC binds CLDN18.2 through its antibody moiety, is internalized, and releases its cytotoxic payload within lysosomes after linker cleavage. A recent study identified autophagy activation as a key mechanism driving ADC resistance in GC cells (**Figure 5**) (75). After ADC exposure, GC cells activate cytoprotective autophagy via Akt/mTOR inactivation. This autophagic response limits ADC-induced apoptosis, and inhibiting autophagy markedly enhances the cytotoxicity of CLDN18.2-directed ADCs *in vitro* and *in vivo*.

ADCs also exert a bystander effect, in which cytotoxic payloads diffuse from targeted cells into neighboring antigen-negative cells. In tumors with heterogeneous CLDN18.2 expression, this can partly overcome the survival of antigen-negative populations (36). The magnitude of the effect depends closely on the physicochemical properties of the payload. High drug-to-antibody ratio (DAR) designs enhance the bystander effect but also increase on-target, off-tumor toxicity, so a balance between efficacy and tolerability is required (5). The site-specific conjugation design of IBI343, with a DAR of 4, showed lower gastrointestinal toxicity in Phase 1 and represents one current direction for ADC optimization (33). Two ADC combination strategies follow from these mechanisms. The first adds an autophagy inhibitor to block intracellular payload clearance (75), and the second adds an ICI to exploit immunogenic cell death (ICD) (76).

The CLDN18.2-ADC pipeline is currently at a stage of development analogous to the early-2010s HER2-ADC landscape, and the HER2 payload-evolution lesson is directly applicable. Trastuzumab emtansine (T-DM1), an anti-HER2 antibody conjugated via a stable linker to DM1, a microtubule-inhibitory maytansine derivative, demonstrated significant clinical benefit in HER2-positive advanced breast cancer in EMILIA, with progression-free survival of 9.6 vs 6.4 months (HR 0.65, 95% CI 0.55–0.77; P < 0.001) and overall survival of 30.9 vs 25.1 months (HR 0.68, 95% CI 0.55–0.85; P < 0.001) versus lapatinib plus capecitabine (77). EMILIA, however, enrolled only centrally confirmed HER2-positive disease (IHC 3+ or FISH ratio ≥ 2.0) and did not address HER2-low tumors. A decade later, trastuzumab deruxtecan (T-DXd), built on a cleavable tetrapeptide-based linker, a topoisomerase-I inhibitor payload, and a drug-to-antibody ratio of approximately 8, delivered transformative outcomes both in HER2-positive gastric cancer (DESTINY-Gastric01: ORR 51% vs 14%, P < 0.001; OS 12.5 vs 8.4 months, HR 0.59, 95% CI 0.39–0.88, P = 0.01) (78) and, decisively for the payload-class argument, in HER2-low metastatic breast cancer (DESTINY-Breast04: PFS HR 0.50; OS HR 0.64, with median OS 23.4 vs 16.8 months in the overall population) (79). DESTINY-Breast04 invoked, rather than itself established, a bystander-effect mechanism, originally described in preclinical work by Ogitani and colleagues, in which the membrane-permeable payload diffuses to neighboring tumor cells that express HER2 heterogeneously, and explicitly supported redefinition of subgroups within HER2-negative breast cancer (79). Applied to CLDN18.2, the current pipeline divides cleanly along this lineage: MMAE-payload agents (AZD0901/CMG901, LM-302; microtubule-inhibitor lineage) likely face a heterogeneity ceiling analogous to that limiting T-DM1, while exatecan-derivative agents (IBI343; topoisomerase-I lineage, DAR 4, site-specific conjugation) plausibly show superior activity in CLDN18.2-low or heterogeneous-expression tumors and against MMAE-resistant clones. This payload-class distinction is more predictive of differential clinical efficacy than total DAR or linker chemistry alone, and classifying CLDN18.2-ADCs by payload class rather than by sponsor may better anticipate their clinical behavior.

### 4.6 Resistance specific to bispecific and multispecific antibodies

Bispecific antibodies (BsAbs) targeting CLDN18.2 are mechanistically distinct from both monoclonal antibodies and CAR-T cells. They are mainly CLDN18.2 by CD3 T-cell redirectors and CLDN18.2 by 4-1BB co-stimulatory activators. Because they are distinct, their resistance profiles warrant dedicated analysis rather than extrapolation from either other modality.

#### 4.6.1 T-cell exhaustion under chronic CD3-redirected engagement

CD3-redirecting BsAbs deliver tonic, TCR-like signaling without classical co-stimulation. Unlike CAR-T cells, which carry engineered co-stimulatory domains, BsAbs engage polyclonal peripheral T cells whose co-stimulatory state is set entirely by the TME. In a co-stimulation-deficient TME, prolonged engagement upregulates the exhaustion regulators TOX and NR4A1 and depletes perforin and granzyme B (80, 81), producing a terminally exhausted CD8 PD-1-high TIM3+ CTLA4+ phenotype largely refractory to PD-1 blockade (81, 82). Because BsAbs engage the entire circulating T-cell pool rather than a defined product, this exhaustion may arise faster and across a broader repertoire than with CAR-T (83), arguing for intermittent dosing or combination with 4-1BB agonism.

#### 4.6.2 CLDN18.2 antigen modulation under BsAb pressure

Whereas conventional antibodies act through ADCC, CD3-redirecting BsAbs require sustained surface CLDN18.2 to maintain immune synapses, yet continuous engagement drives profound antigen modulation through rapid internalization and lysosomal degradation. Bivalent CLDN18.2–CD3 crosslinking promotes cooperative clustering and co-internalization that may outpace mAb-driven loss, as documented for CD19-directed BsAbs (84, 85); surviving clones may further downregulate claudin-regulatory pathways toward durable antigen negativity. BsAb therapy therefore amplifies rather than mitigates antigen heterogeneity, making serial CLDN18.2 assessment after treatment even more important than after mAb therapy.

#### 4.6.3 Lessons from CAR-T and bispecific precedents for combination and dosing

The CD3-engaging exhaustion phenotypes described above have direct antecedents in the CAR-T and bispecific-antibody literature that inform combination and dosing strategy along three lines: tonic signaling and costimulation, delivery and trafficking, and dosing schedule. (i) Tonic signaling and costimulation. The foundational observation that tonic, antigen-independent CAR signaling, driven by scFv framework-mediated clustering and aggregation on the T-cell surface, induces early T-cell exhaustion through chronic CD3-ζ phosphorylation was made for the GD2-targeted CAR, with CD28 costimulation aggravating and 4-1BB costimulation ameliorating the phenotype (86). This early work provided the biological rationale, namely scFv-framework engineering and the choice of costimulatory domain, that informed subsequent CAR design. The principle is equally relevant to BsAb format choice, including affinity-modulated CD3 binders [the design rationale for AZD5863 (87)] and conditional 4-1BB co-stimulation (the rationale for givastomig (88) and AZD5863’s intratumor-activation profile). (ii) Delivery and trafficking. The mesothelin CAR-T experience extends this lesson. An mRNA-engineered mesothelin CAR-T administered intravenously to patients with malignant pleural mesothelioma and metastatic pancreatic cancer was feasible and trafficked to primary and metastatic tumor sites but persisted only transiently in peripheral blood, with antibody responses against the murine scFv producing anaphylaxis and limiting repeat dosing; this was among the first demonstrations of systemic-delivery feasibility and its limits (89). A subsequent phase 1 trial of regional intrapleural mesothelin CAR-T plus pembrolizumab in malignant pleural disease delivered prolonged survival (median OS 23.9 months from CAR-T infusion in the combination subset; 1-year OS 83%) (90). The investigators’ rationale for regional rather than systemic delivery was twofold: the locoregional aggressiveness of mesothelioma, and orthotopic-model evidence that regional administration avoided CAR-T sequestration in lungs and augmented CD4 helper function. They further noted that solid-tumor T-cell infiltration, antigen heterogeneity, and an immunosuppressive microenvironment together constrain systemic CAR-T efficacy (90). (iii) Dosing schedule and biomarkers. Two concrete implications follow for CLDN18.2 BsAb programs. First, any CLDN18.2-CD3 BsAb in pancreatic or diffuse-type gastric trials should be tested with intermittent dosing schedules from the start, drawing on blinatumomab treatment-free-interval data that demonstrated functional reinvigoration and transcriptional reprogramming of exhausted T cells (day-14 specific lysis 93.4% with treatment-free intervals vs 34.9% with continuous exposure) (83). Second, trafficking biomarkers (pre- and on-treatment intratumoral CD8 density, peripheral CXCL12 dynamics, and CAR-T or BsAb persistence on serial samples) should be incorporated as pre-specified pharmacodynamic endpoints in BsAb solid-tumor trials, where they have so far been absent or *post-hoc*.

## 5 Clinical countermeasures against resistance

### 5.1 Dynamic biomarker monitoring and personalized management

Because of significant spatiotemporal heterogeneity and dynamic treatment-induced changes, a single baseline biopsy cannot reliably guide CLDN18.2-targeted therapy: intratumoral heterogeneity occurs in 38.5% of cases (27) and 40% of patients lose expression after one chemotherapy cycle (28). This also challenges RECIST, since a shrinking lesion may still harbor CLDN18.2-negative resistant clones, while apparent progression may reflect immunotherapy- or CAR-T-induced pseudoprogression rather than true growth. The distinction is especially difficult when CRS-associated edema transiently enlarges tumors on CT. Two emerging tools can help.

The first is ctDNA liquid biopsy. Longitudinal ctDNA should be incorporated as a pharmacodynamic biomarker alongside RECIST: a molecular response preceding radiographic change may predict durable benefit, whereas molecular progression without RECIST change may flag emerging resistance and warrant biopsy-guided adjustment. Standardization remains difficult because CLDN18.2 is regulated transcriptionally and post-translationally (2), and promoter methylation is a candidate ctDNA resistance marker still requiring analytical validation (91).

The second is CLDN18.2-targeted molecular PET, which enables whole-body, lesion-by-lesion antigen mapping and directly addresses biopsy sampling bias (92, 93). An early SUV reduction may indicate antigen downregulation before RECIST progression, and dual-tracer imaging combining 18F-FDG with CLDN18.2-PET could distinguish inflammatory pseudoprogression from true antigen-loss resistance; because CLDN18.2-positive tumors tend to show lower baseline FDG uptake, standard thresholds may be inappropriate here (94, 95).

For phase 3 trials specifically, we propose a tiered biomarker scheme. The first tier directly tracks target dynamics: serial on-treatment CLDN18.2 re-biopsy by standardized IHC together with whole-body CLDN18.2-PET (92, 93), which quantify the depleting target lesion by lesion. The second tier provides early molecular readout through longitudinal ctDNA, with CLDN18 promoter methylation as a candidate resistance marker requiring analytical validation (91). The third tier reads out the resistance microenvironment: the serum kynurenine-to-tryptophan ratio (68), peripheral CXCL12 and CAR-T or bispecific persistence, and baseline NLR (70). We recommend these be embedded as pre-specified, longitudinal, co-primary pharmacodynamic endpoints.

Conventional scoring of CLDN18.2, based on manual estimation of at least 75% of tumor cells at 2+/3+, is subjective and vulnerable to intratumoral heterogeneity and sampling bias (7, 27). Whole-slide digital pathology with AI-assisted quantification could provide continuous, spatially resolved and reproducible heterogeneity metrics, for example mapping the fraction and topology of CLDN18.2-retaining regions, and may better predict which tumors carry a genuine, durable targeting advantage. As an early proof of concept, artificial-intelligence models applied to routine histology can already infer CLDN18.2 expression and immune phenotype to help guide treatment selection (96). These tools remain exploratory and require prospective, multi-reader validation against outcome before they can refine patient selection.

There is an urgent need for a consensus response-assessment framework tailored to CLDN18.2-targeted therapy. Such a framework would incorporate molecular imaging, longitudinal ctDNA monitoring, and biopsy-triggered reassessment criteria, and it should be a primary objective for ongoing Phase 3 trial consortia.

### 5.2 Combination strategies targeting the immunosuppressive TME

Rational TME-directed combinations can be grouped into four orthogonal, mechanism-anchored strategies, each tied to a resistance node identified above: (i) the tryptophan-kynurenine/IDO1 axis, combining an IDO1 inhibitor with CLDN18.2 CAR-T (68), tempered by the ECHO-301 cautionary experience (97); (ii) the CXCR4/CXCL12 axis, using a CXCR4 antagonist (73) or CXCR4-modified CAR-T (98) to counter MDSC recruitment and the β-catenin/CXCL12 marrow-homing circuit (60); (iii) TGF-β/Treg/stromal targeting, with TGF-β-neutralizing antibodies or CTLA-4 inhibition in the high-Treg, fibrotic subgroup (28, 67); and (iv) neutrophil-directed intervention, with baseline NLR as a predictor and targeting of NE-3-associated pathways (70). As argued above, the platform-level lesson from stroma-rich tumors (72–74) is that any added TME agent should function as a niche-clearing adjuvant to a CLDN18.2-directed cytotoxic backbone rather than as stand-alone immunotherapy. The specific combinations below are organized within this framework.

Several combinations follow directly from the resistance mechanisms described above. Based on the IDO1 and kynurenine pathway, combining an IDO1 inhibitor with CLDN18.2 CAR-T has strong translational potential, having been validated preclinically (68). Based on NE-3 neutrophil-mediated resistance, monitoring baseline NLR may serve as a practical predictive tool, and interventions targeting NE-3-characteristic pathways may improve CAR-T response rates (70). For Treg and TGF-β-mediated immunosuppression, combination with TGF-β-neutralizing antibodies or CTLA-4 inhibitors warrants exploration, particularly for CLDN18.2-positive patients who have high baseline Treg infiltration and a fibrotic stroma (28).

The preclinical rationale for IDO1 + CLDN18.2 CAR-T (68) must be assessed against the field’s most prominent IDO1 clinical trial. ECHO-301/KEYNOTE-252 tested epacadostat 100 mg orally twice daily plus pembrolizumab 200 mg IV every 3 weeks against placebo plus pembrolizumab in 706 patients (354 vs 352) with unresectable stage III or IV melanoma previously untreated with PD-1/PD-L1 inhibitors. The trial showed no improvement in progression-free survival (median 4.7 vs 4.9 months; HR 1.00, 95% CI 0.83–1.21; one-sided P = 0.52) or overall survival (median not reached in either group; HR 1.13, 95% CI 0.86–1.49; one-sided P = 0.81), and was stopped after the second interim analysis on the recommendation of the external independent data monitoring committee (97). Although the impact on subsequent IDO1 Phase 3 programs is widely reported in the broader literature and field commentary, ECHO-301 itself does not enumerate those downstream discontinuations, and we cite it here only for what its own data demonstrate.

Three lessons from this failure are directly relevant to CLDN18.2 trial design. First, the combination context matters: epacadostat was paired with pembrolizumab in melanoma, the ICI's monotherapy activity. CLDN18.2 CAR-T and BsAb monotherapy in solid tumors is more modest [Phase 1 ORR ~30–40% for satri-cel (31); lower for current BsAbs (99)], leaving more measurable upside for an effective adjuvant but not exempting IDO1 combinations from the requirement of demonstrating pharmacodynamic effect. Second, the trial itself identified mechanistic concerns: the 100 mg twice-daily dose may have produced incomplete IDO1 target coverage, and IDO1 expression in melanoma is markedly heterogeneous between and within patients longitudinally (97); future CLDN18.2 + IDO1 trials should pre-specify dual-pathway inhibitors (targeting TDO2 and IDO2 in parallel) where feasible, or at minimum measure TDO2 expression and kynurenine kinetics as pharmacodynamic correlates. Third, on-treatment pharmacodynamic biomarkers are essential: the serum kynurenine-to-tryptophan ratio is measurable, dynamic, and mechanistically anchored, and should be incorporated as a pre-specified PD biomarker in any CLDN18.2 + IDO1 Phase 1b with demonstrated kynurenine suppression required before advancing to randomized Phase 2. Under these design constraints the CLDN18.2 + IDO1 rationale remains worth testing, particularly in the CAR-T setting where lymphodepletion-induced reduction of tumor IDO1 (68) creates a transient permissive niche, but the field cannot proceed as though ECHO-301 did not happen.

### 5.3 Multi-target combination therapy

Rational combinations for CLDN18.2-targeted therapy fall into three mechanism-anchored categories, each matched to one axis of the resistance map: (i) restoring or exploiting anti-tumor immunity, pairing a CLDN18.2 agent with an immune checkpoint inhibitor guided by PD-L1/EBV/MSI status; (ii) neutralizing the immunosuppressive stroma, through TGF-β, CXCR4, or IDO1 blockade (Section 5.2); and (iii) countering antigen heterogeneity and escape, through ADC bystander payloads, dual-antigen constructs, and induction-then-maintenance sequencing (Section 5.4). The subtype-specific examples below are organized within this framework, beginning with checkpoint-inhibitor combinations, for which the most mature comparative evidence exists.

About 30% of CLDN18.2-positive tumors co-express PD-L1 (CPS at least 1) (100), so optimizing the combination and sequencing of zolbetuximab with ICIs, beyond a CPS threshold alone, remains pivotal. EBV-positive GC (53), is a setting where ICI-based regimens may build a synergistic TME for zolbetuximab-mediated ADCC. In MSI-H/dMMR tumors (about 4 to 7% of advanced GC), PD-1 blockade is the anchor, and adding zolbetuximab after ICI induction may prevent CLDN18.2 escape during clonal selection. The predominant EBV-negative, MSS subtype in SPOTLIGHT (16) and GLOW (17) shows RHOA activation (65), high Treg/TGF-β infiltration and a fibrotic stroma (28) that both blocks chemotherapy penetration and mediates ICI resistance; here zolbetuximab plus chemotherapy is the mechanistically justified backbone, and whether added pembrolizumab (NCT06901531) helps may hinge on chemotherapy transiently disrupting the fibrotic barrier.

These subtype–pathway correlations warrant biomarker-stratified analyses: EBV and MSI-H tumors engage immunosuppressive microenvironments, whereas MSS tumors exhibit antigen heterogeneity and polarity barriers, so population-level OS may obscure cohort-specific needs. Current evidence favors zolbetuximab at PD-L1 CPS below 10 and ICIs at CPS at least 10 (101), a boundary the Phase 3 triplet (NCT06901531) will test directly. ADC–ICI combinations such as LM-302 plus tislelizumab plus CAPOX and an AZD0901 multi-tumor Phase 2 (102) probe ADC-induced immunogenic cell death, especially relevant in MSS tumors. CLDN18.2/HER2 co-expression occurs in a GC subset (103), and the dual-targeting bispecific HC-2G4S outperformed single agents and a two-drug combination *in vivo* (102), a proof of concept for dual-target strategies.

The triplet hypothesis advanced by LUCERNA (NCT06901531), that zolbetuximab + pembrolizumab + chemotherapy will outperform either chemoimmunotherapy or chemo + zolbetuximab in CLDN18.2-positive, HER2-negative advanced GC/GEJC, must be interpreted alongside the KEYNOTE-859 evidence base. KEYNOTE-859 tested pembrolizumab 200 mg IV every 3 weeks plus investigator’s choice of fluoropyrimidine-platinum chemotherapy (5-FU + cisplatin or capecitabine + oxaliplatin) versus placebo plus chemotherapy in 1,579 patients (790 vs 789) with previously untreated, HER2-negative, locally advanced unresectable or metastatic gastric or gastro-esophageal junction adenocarcinoma, and met its primary OS endpoint in the ITT population (median 12.9 vs 11.5 months; HR 0.78, 95% CI 0.70–0.87; P < 0.0001) (104). A pre-specified PD-L1 CPS-stratified analysis revealed substantial heterogeneity of benefit: in patients with CPS ≥ 10 the stratified OS HR was 0.65 (95% CI 0.53–0.79; median 15.7 vs 11.8 months); in a *post-hoc* unstratified analysis of CPS 1–9 patients the OS HR was 0.83 (95% CI 0.70–0.98), with an upper bound that did not cross unity although the analysis was exploratory and underpowered; and in the CPS < 1 subgroup (unstratified) the OS HR was 0.92 (95% CI 0.73–1.17), with the CI crossing unity and acknowledged by the authors as a small, exploratory subgroup (104). The CPS < 1 stratum thus showed no demonstrable benefit from pembrolizumab, and the CPS 1–9 stratum showed only marginal benefit on a *post-hoc* basis.

The implication for LUCERNA is direct. The trial’s CLDN18.2-positive, PD-L1 CPS-positive enrollment criterion will recruit patients across the CPS 1–10 and CPS ≥ 10 strata. The marginal incremental benefit of adding pembrolizumab to a zolbetuximab + chemotherapy backbone will likely concentrate in the CPS ≥ 10 subgroup, where ICI contribution is well-established, and may be modest or negligible in the CPS 1–9 stratum where KEYNOTE-859 itself shows only a *post-hoc* exploratory signal. We recommend that LUCERNA pre-specify both: (i) a CPS-stratified primary analysis with a pre-defined futility rule for the CPS 1–9 stratum at planned interim, allowing early termination of unwarranted exposure to pembrolizumab-related immune toxicity in patients unlikely to benefit; and (ii) a fallback regimen recommendation of zolbetuximab + chemotherapy alone for the CPS 1–9 stratum if the triplet does not deliver incremental benefit. Conducting LUCERNA without such CPS-aware design risks repeating the KEYNOTE-859 averaging effect, in which an overall positive ITT result masks subgroup-level heterogeneity and the field defaults to triplet therapy for patients who do not gain from the additional agent.

### 5.4 Treatment sequencing: induction intensification and CLDN18.2 maintenance

A recurring theme across the resistance mechanisms above is that CLDN18.2 is not a static target but a depleting one. Treatment-induced antigen loss is rapid and frequent: in serial-biopsy studies, roughly 40% of initially positive gastric tumors lost CLDN18.2 expression after a single chemotherapy cycle, spatiotemporal heterogeneity is pervasive, and antibody-, ADC-, and bispecific-driven pressure each select for antigen-low clones. A continuous target-plus-chemotherapy regimen administered to progression therefore operates against a substrate that erodes over the very interval the regimen is meant to control, with antibody-dependent cytotoxicity, ADC payload delivery, and immune-synapse formation all weakening as surface CLDN18.2 falls. This kinetic mismatch, in which maximal drug exposure is maintained while target density declines, may be an under-recognized contributor to the eventual failure of CLDN18.2-directed regimens, including in patients selected for high baseline expression.

We propose that, for patients with a genuine CLDN18.2-targeting advantage, treatment may be better structured as induction and maintenance rather than as a single continuous regimen carried to progression. During the early, treatment-naïve window, when target density is highest and most homogeneous, an intensive, time-limited induction combining chemotherapy with a CLDN18.2-directed agent, and where the biology supports it an immune checkpoint inhibitor or an ADC, would exploit maximal antigen availability to achieve the deepest possible cytoreduction. Once maximal response is reached, the cytotoxic backbone, which is the principal driver of both antigen attrition and cumulative toxicity, could be de-escalated, and disease control sustained with CLDN18.2-directed maintenance monotherapy, such as a monoclonal antibody or a well-tolerated ADC, in the residual, antigen-retaining disease. This mirrors the induction–consolidation–maintenance architecture that improved outcomes in other antigen- and pathway-targeted malignancies, and it reframes the CLDN18.2 antibody not as a perpetual chemotherapy partner but as a maintenance agent deployed after the high-antigen window has been used to greatest effect.

Defining the CLDN18.2-targeting advantage that would justify this approach requires more than a single positive biopsy: candidate criteria include high and homogeneous baseline expression (2+/3+ in at least 75% of cells), HER2 negativity, and, ideally, demonstrated antigen retention on early on-treatment re-biopsy or molecular imaging. Several caveats apply. Maintenance-monotherapy efficacy after induction is unproven for CLDN18.2 and must be established prospectively; single-agent maintenance could itself select for antigen-negative escape, arguing for antigen-monitoring-guided maintenance or a low-intensity combination backbone; and the optimal induction composition, duration, and response threshold for transition all remain open questions. Nonetheless, the proposal generates clear, testable hypotheses. Most directly, a fixed-duration intensive induction followed by antibody maintenance would be predicted to yield superior progression-free survival and tolerability versus continuous target-plus-chemotherapy in high-expression patients, and we suggest that on-treatment antigen dynamics be incorporated as a co-primary pharmacodynamic endpoint in the trials that test it.

## 6 Pan-cancer perspective: context-dependent biology of CLDN18.2

**Figure 6:**
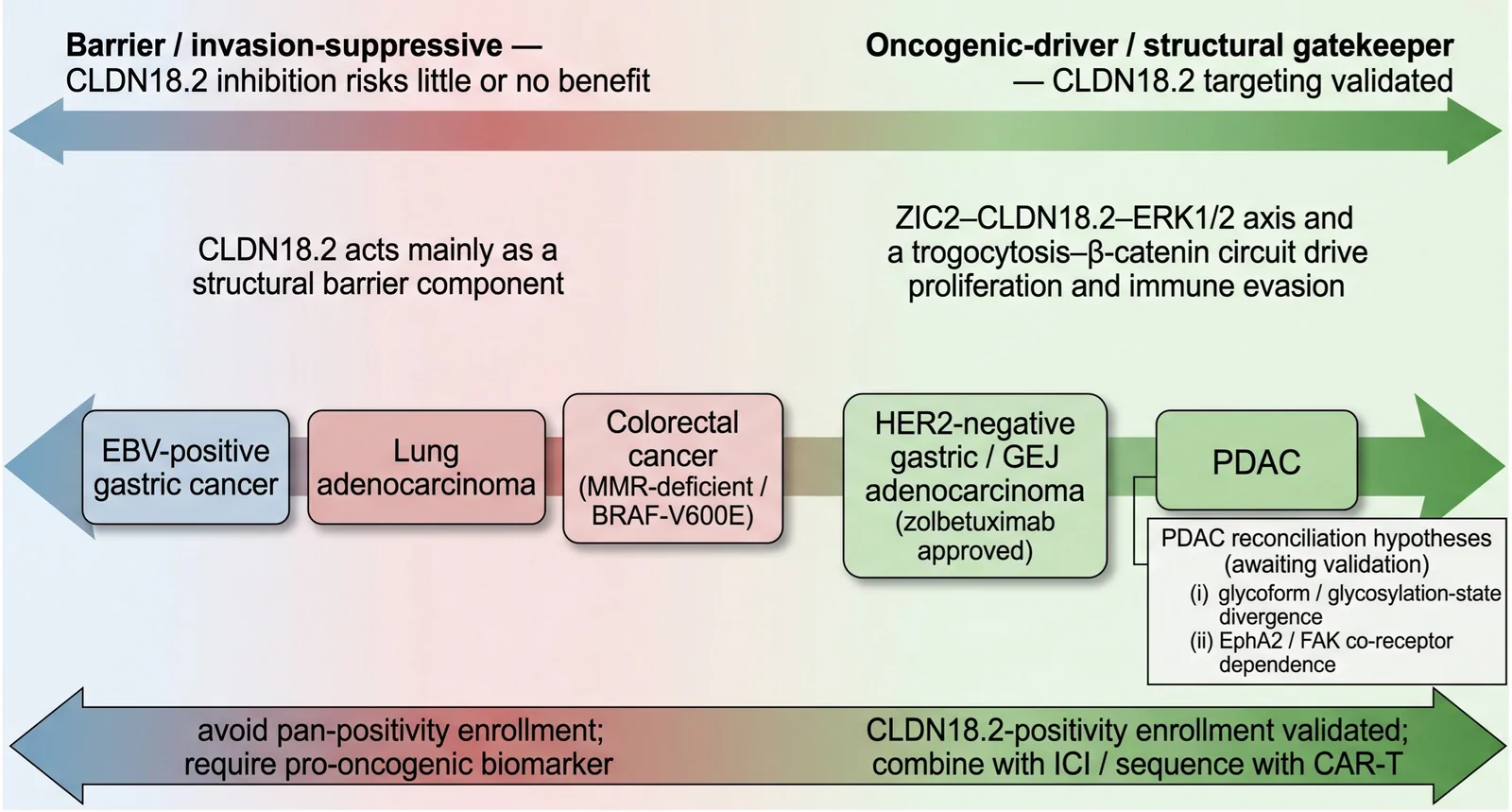
Context-dependent functional polarity of CLDN18.2 across tumor types.

Beyond the temporal sequencing argument advanced above, a complementary consideration for patient selection is that CLDN18.2 biology is not uniform across tumor types. Across cancers, CLDN18.2 exhibits tissue-specific functional polarization (**Figure 6**), with at times opposing roles set by cellular context (46), the co-receptor landscape (105), and post-translational modifications (106). In HER2-negative gastric and gastro-esophageal junction adenocarcinoma the protein is a high-yield therapeutic target and the molecular basis for zolbetuximab approval. In lung adenocarcinoma and in subsets of pancreatic ductal adenocarcinoma, however, the available evidence suggests CLDN18.2 may instead mark an invasion-suppressive epithelial state (42, 107), so that the magnitude, and even the direction, of benefit from targeting it could vary by tissue of origin. This section surveys that context-dependent biology.

In gastric cancer, high CLDN18.2 expression associates with diffuse-type (Lauren) histology (108). EBV-positive tumors (53), represent an immune-hot subtype in which CLDN18.2 acts mainly as a structural component rather than an oncogenic driver. Zolbetuximab efficacy here likely reflects ADCC-mediated elimination of well-differentiated, immunologically accessible clones, with resistance driven by the immunosuppressive TME; this supports ICI plus zolbetuximab combinations in EBV-positive, CLDN18.2-positive GC.

In PDAC, CLDN18.2 presents a functional paradox. Clinically, high expression predicts superior OS and recurrence-free survival after resection, consistent with a well-differentiated, less invasive phenotype (107); yet molecularly the ZIC2-CLDN18.2-ERK1/2 axis promotes proliferation, migration and EMT in cell lines and TCGA data (48). This discrepancy may reflect different processing states of one protein: a fully glycosylated, junction-resident pool that maintains the barrier and tracks with favorable prognosis, versus hypoglycosylated or co-receptor-engaged forms, for example with EphA2 or FAK, that translocate and drive ERK1/2 signaling, such that the net effect of targeting CLDN18.2 in any given PDAC may depend on which pool predominates (48, 106, 109). This remains a hypothesis awaiting experimental validation.

In lung adenocarcinoma, CLDN18.2 positivity is only about 8%, yet strong positivity predicts better prognosis, particularly in EGFR-wild-type, PD-L1-below-1% double-negative tumors (42). This inverse relationship may reflect an invasion-constraining polarity, with such tumors retaining alveolar differentiation and lower invasive potential. Whether targeting CLDN18.2 provides net benefit in this context is therefore uncertain, and clinical evaluation will warrant both robust activity data and careful attention to disease behavior in early-phase trials.

About 3% of colorectal cancers are CLDN18.2-positive, enriched in MMR-deficient and BRAF V600E-mutated tumors (11) that already benefit from ICIs or BRAF/MEK inhibition, so the added value of CLDN18.2 targeting needs validation. Overall, the impact of CLDN18.2-targeted therapy is shaped by this context-dependent functional polarity: whether CLDN18.2 acts predominantly as an epithelial gatekeeper (46), an immunomodulatory anchor, or a signaling scaffold may help explain the differing outcomes observed across tumor types (48). Considering tumor-type context alongside expression level, rather than treating positivity as a uniform biomarker, is therefore a useful adjunct to patient selection.

## 7 Discussion: resistance landscape and research priorities

CLDN18.2-targeted therapy has moved from target discovery and approval into a phase defined by resistance dissection and precision-combination design. As detailed in Sections 4 and 5, resistance is best understood not as a linear pathway but as a networked landscape of six mutually reinforcing dimensions. Because these nodes reinforce one another, monotherapy rarely disrupts them completely. Across these dimensions, the most actionable single insight is that the target itself is dynamic: CLDN18.2 density declines under treatment pressure, which motivates the induction-and-maintenance sequencing strategy proposed earlier and frames the priorities below. Rather than restating each mechanism, this section distills four cross-cutting priorities that follow from them.

First, dynamic, multimodal monitoring, including ctDNA tracking extended to CLDN18.2 promoter-methylation dynamics (2, 91), whole-body CLDN18.2-PET (92, 93), and dual-tracer imaging to separate antigen-loss resistance from pseudoprogression (94, 95), should replace the one-biopsy paradigm, ideally formalized as a CLDN18.2-specific, iRECIST-style framework in Phase 3 consortia. Second, systemic immunosuppression must be addressed: the trogocytosis–β-catenin–marrow-senescence axis (60), although based on a single foundational study and pending independent replication, prioritizes validation via marrow sampling and exploratory testing of CXCR4 antagonists and the PC18.1 peptide, while the treatment-induced IDO1–kynurenine axis argues for on-treatment monitoring and pre-emptive IDO1 inhibition in BsAb and CAR-T regimens (68, 110). Third, next-generation engineering, including AND-gate (64), IL-7/XCL1-armored (111) and trispecific or PD-1-converting constructs (112, 113), aims to resolve toxicity, exhaustion and immune exclusion, with exhaustion kinetics and antigen-modulation behavior the decisive open questions (83, 85). Fourth, combinations should be mechanism-matched: ADC plus an autophagy inhibitor or an ICI (75), and biomarker-stratified triplets for EBV/MSI-H versus MSS disease (16, 17, 28, 53, 98).

As in any review of a rapidly evolving field, several considerations frame the conclusions drawn here; for each, we note the step taken to address it. First, because CLDN18.2-targeted therapy is early in its clinical maturation, part of the evidence comes from early-phase trials and translational studies and cross-modality head-to-head data are not yet available; we have therefore labeled every claim by its level of evidence and separated established mechanisms from proposed ones. Second, several of those proposals, namely the cell-polarity chemoresistance barrier, the glycoform and co-receptor models in pancreatic cancer, and the induction-and-maintenance sequencing strategy central to this review, are not yet experimentally confirmed; rather than present them as fact, we flag each explicitly and pair it with a specific, testable prediction and a candidate pharmacodynamic endpoint such as on-treatment antigen dynamics, so that it can be evaluated in trials. Finally, we adopted a narrative rather than systematic format by design, to integrate mechanistic, translational and clinical evidence across four therapeutic modalities within a single synthesis; to limit the attendant selection bias, we drew efficacy and safety figures directly from the primary trial reports and organized the resistance literature along an explicit six-dimension framework rather than *ad hoc*. The prospective validation these points call for is, in our view, the roadmap this synthesis is intended to generate rather than a shortcoming of it.

Equity and access considerations. A separate set of considerations relates to the equitable global deployment of CLDN18.2-targeted therapy. Most pivotal CLDN18.2 trials to date are industry-sponsored and concentrated among a small number of developers in China, the United States and Europe. This concentration, together with the regional clustering of early-phase trials, makes broad multi-regional validation and timely global access important goals; differential regulatory pathways and pricing may otherwise widen access gaps even after registration. The companion-diagnostic ecosystem currently depends on a single immunohistochemistry assay (VENTANA CLDN18 43-14A), which limits laboratory choice and creates supply-concentration risk; emerging alternative clones and PET-based functional assays merit parallel development. Finally, the cost of stacking a CLDN18.2-ADC with checkpoint blockade, or of autologous CAR-T with lymphodepletion, may limit deployment in community-oncology and lower- and middle-income settings without coordinated payer engagement. We flag these as priorities for parallel policy and health-systems work.

In sum, the field’s next decade will be defined less by the number of CLDN18.2-directed agents than by how they are deployed. Treating CLDN18.2 positivity as a uniform, static biomarker is no longer adequate: the timing, intensity, and tissue context of therapy must instead be matched to the dynamic, context-dependent biology of the target. Realizing this will require the prospective, biomarker-embedded trials outlined here, but it offers the most promising route to durable and broadly accessible benefit across CLDN18.2-positive malignancies.
